# Array-Based Discriminative Optical Biosensors for Identifying Multiple Proteins in Aqueous Solution and Biofluids

**DOI:** 10.3389/fchem.2020.572234

**Published:** 2020-11-04

**Authors:** Junmei Fan, Lu Qi, Hongfei Han, Liping Ding

**Affiliations:** ^1^Department of Chemistry, Taiyuan Normal University, Jinzhong, China; ^2^Key Laboratory of Applied Surface and Colloid Chemistry, Ministry of Education, School of Chemistry and Chemical Engineering, Shaanxi Normal University, Xi'an, China

**Keywords:** protein, sensor array, nanoparticles, amphiphilic aggregate, environment-sensitive system

## Abstract

Identification of proteins is an important issue both in medical research and in clinical practice as a large number of proteins are closely related to various diseases. Optical sensor arrays with recognition ability have been flourished to apply for distinguishing multiple chemically or structurally similar analytes and analyzing unknown or mixed samples. This review gives an overview of the recent development of array-based discriminative optical biosensors for recognizing proteins and their applications in real samples. Based on the number of sensor elements and the complexity of constructing array-based discriminative systems, these biosensors can be divided into three categories, which include multi-element-based sensor arrays, environment-sensitive sensor arrays and multi-wavelength-based single sensing systems. For each strategy, the construction of sensing platform and detection mechanism are particularly introduced. Meanwhile, the differences and connections between different strategies were discussed. An understanding of these aspects may help to facilitate the development of novel discriminative biosensors and expand their application prospects.

## Introduction

Proteins are fundamental components of cells and tissues of human bodies, and play important roles in various life processes, such as repairing tissue, transporting substances and maintaining the normal metabolism (Zhao et al., 2016; Wang et al., 2019). Studies have reported that proteins are involved in diagnosis of various affiliated diseases including Alzheimer's, Parkinson's, Huntington's, and prion diseases (Galdeano et al., 2012; Scala et al., 2013). Abnormal protein concentration levels in biofluids (serum, urine, etc.), cells or tissues can provide necessary information for the early diagnosis of various pathological conditions (Li and Liu, 2010; Kong et al., 2011). Thus, it is of great significance to quantitatively analyze and specially recognize proteins for applications in medical diagnostics, proteomics and pathogen detection.

Among many detection methods for proteins, discriminative optical sensors have exhibited advantages like high sensitivity, high-throughput and real-time online detection, which have gained increasing attention (Zhu et al., 2015; Zhang et al., 2017; Fan and Ding, 2018). Besides, such cross-reactive sensors enable the recognition of structurally or chemically similar analytes and even the identification of mixed or unknown samples (Lin et al., 2016; Bigdeli et al., 2017; Wang et al., 2018). One way to achieve discriminative sensing is to develop multi-element sensor arrays, which are inspired by mimicking mammalian taste and smell systems and usually consist of multiple cross-reactive elements that generate a combined recognition pattern for each analyte (Stewart et al., 2013; Peveler et al., 2016; Rana et al., 2016). Another way to realize discriminative sensing is to fabricate an environment-sensitive sensor array, which is constructed by changing the solvents (Cao et al., 2014b, 2020; Smith et al., 2019), probe concentrations (Li et al., 2014), or pH values and ionic strengths (Liu et al., 2017; Tomita et al., 2017; Zhou et al., 2017; Lin et al., 2019). The third type of discriminative sensing is multi-wavelength cross-reactive single-system-based sensors, which use multiple wavelengths instead of multi-elements to provide response signals (Wu et al., 2011; Rout et al., 2012). During the past few decades, these three types of optical sensors, especially the first type, have been widely employed for protein discrimination.

In this review, we focus on the array-based discriminative optical biosensors for identifying proteins according to the above-mentioned three strategies. The construction principle, sensing mechanism, sensitivity and accuracy, and practical application (protein identification in serum or urine and cell or bacterium discrimination) of various sensors are particularly introduced in detail. An understanding of these aspects may facilitate the development of novel discriminative optical biosensors and expand their application prospects.

## Multi-Element-Based Sensor Arrays for Protein Recognition

The most widely adopted strategy of building sensor arrays is to use a number of cross-reactive sensors as elements to provide multiple response signals and generate recognition patterns for analytes. A variety of sensor elements like conjugated polymer, amphiphilic aggregates, nanoparticles, quantum dots, etc., have been used to generate multiple element sensor arrays for the purpose of protein identification.

### Conjugated Polymers

Conjugated polymers (CPs) have been extensively explored for chemical and biological sensor design due to their highly delocalized electronic structures and unique optical and electronic properties (Chen et al., 2013; Zhao et al., 2017). The delocalized structures of CP backbones allow efficient intra- and inter-chain energy transfer that amplifies signals by the collective response compared with small molecule-fluorophores. Their optical properties (absorption and emission) are sensitive to minor conformational or environmental variations, enabling efficient detection of subtle differences when bound with various analytes in sensing processes. Water-soluble CPs with hydrophobic backbones and hydrophilic side-chains or ionic units can associate with different proteins through multivalent interactions, producing unique optical responses to different proteins (Feng et al., 2010).

Based on the non-specific interaction between conjugated polymers and proteins, Miranda et al. (2007) reported using six functionalized poly(*p*-phenyleneethynylene)s (PPEs) to create a six-element sensor array for identifying 17 proteins which have diverse molecular weight, metal/non-metal-containing, isoelectric point (pI), and UV absorbencies. LDA results illustrated that the tested 17 proteins could be well-clustered into 17 different groups with a classification accuracy of 100%. Moreover, out of the 68 protein samples randomly selected from 17 proteins, only two samples were misclassified, and the identification accuracy was 97%. This work demonstrated that a PPE-based sensor array could effectively detect and distinguish proteins.

**Figure 1:**
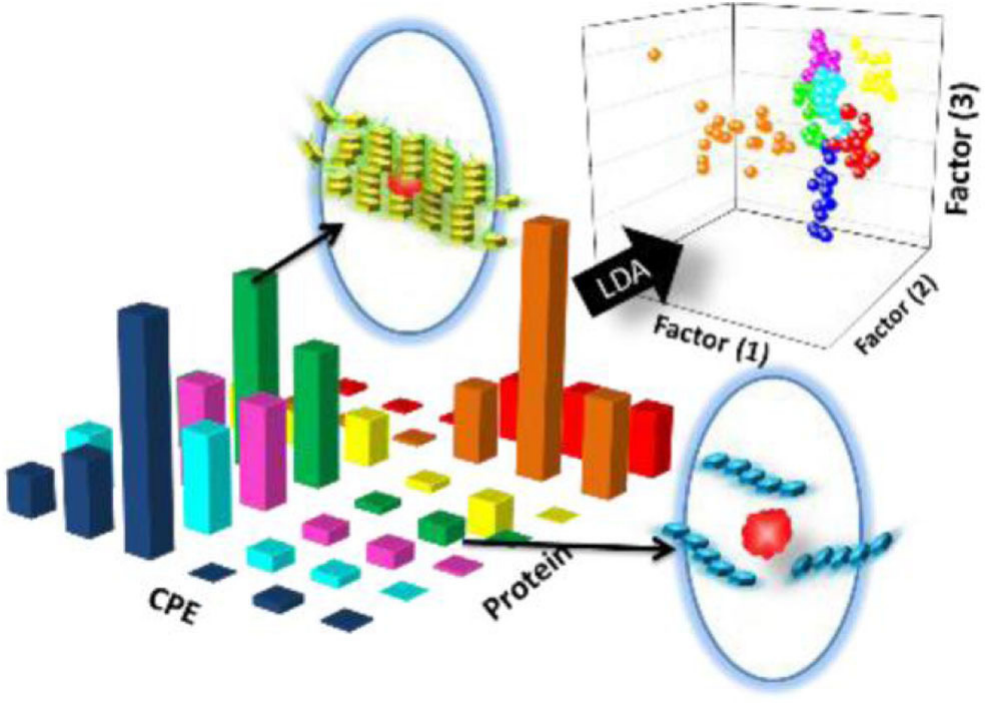
Schematic representation of the CPEs-based sensor array for the discrimination of proteins. Reprinted from Wu and Schanze (2014) with permission.

Wu and Schanze (2014) constructed a fluorescent sensor array containing six conjugated polyelectrolytes (CPEs) and explored the aggregation state/size change upon binding with a set of different proteins by fluorescence correlation spectroscopy (Figure 1). This CPEs-based array could well identify seven proteins and successfully discriminate unknown protein samples with an accuracy of 93% by LDA. They found that the charge type (cation and anion) of CPEs played the most important role in protein recognition compared to other factors, such as charge density, molecular weight, and backbone structure. This kind of probes can be optimized by increasing the purity of polymers, conjugating more diverse functional groups to backbones or introducing novel CPE probes. Importantly, one of the challenges of this type of sensors is the need to eliminate potential interference when used for detection in complex biological environments.

### Amphiphilic Aggregates

As we all know, amphiphilic molecules, such as surfactants, dendrimers, and block copolymers can form dynamic supramolecular aggregates, such as micelles and vesicles in aqueous solutions (Yan et al., 2010, 2012). The strategy of amphiphilic aggregates encapsulating and modulating fluorophores has been widely applied in construction of fluorescent sensors or arrays for proteins because they can provide several advantages: (1) The hydrophobic domains can non-covalently encapsulate guest probes to improve their solubility, fluorescence stability and quantum yield (Hu et al., 2010; Geng et al., 2014; Fan et al., 2019). (2) The subtle variation of amphiphilic molecule aggregation states can modulate the photophysical properties and fluorescence emission of encapsulated probe (Ding et al., 2013; Cao et al., 2014a; Cao J. et al., 2014). Thus, protein-caused aggregation changes of amphiphilic assemblies can further induce fluorescence variation for detecting proteins.

**Figure 2:**
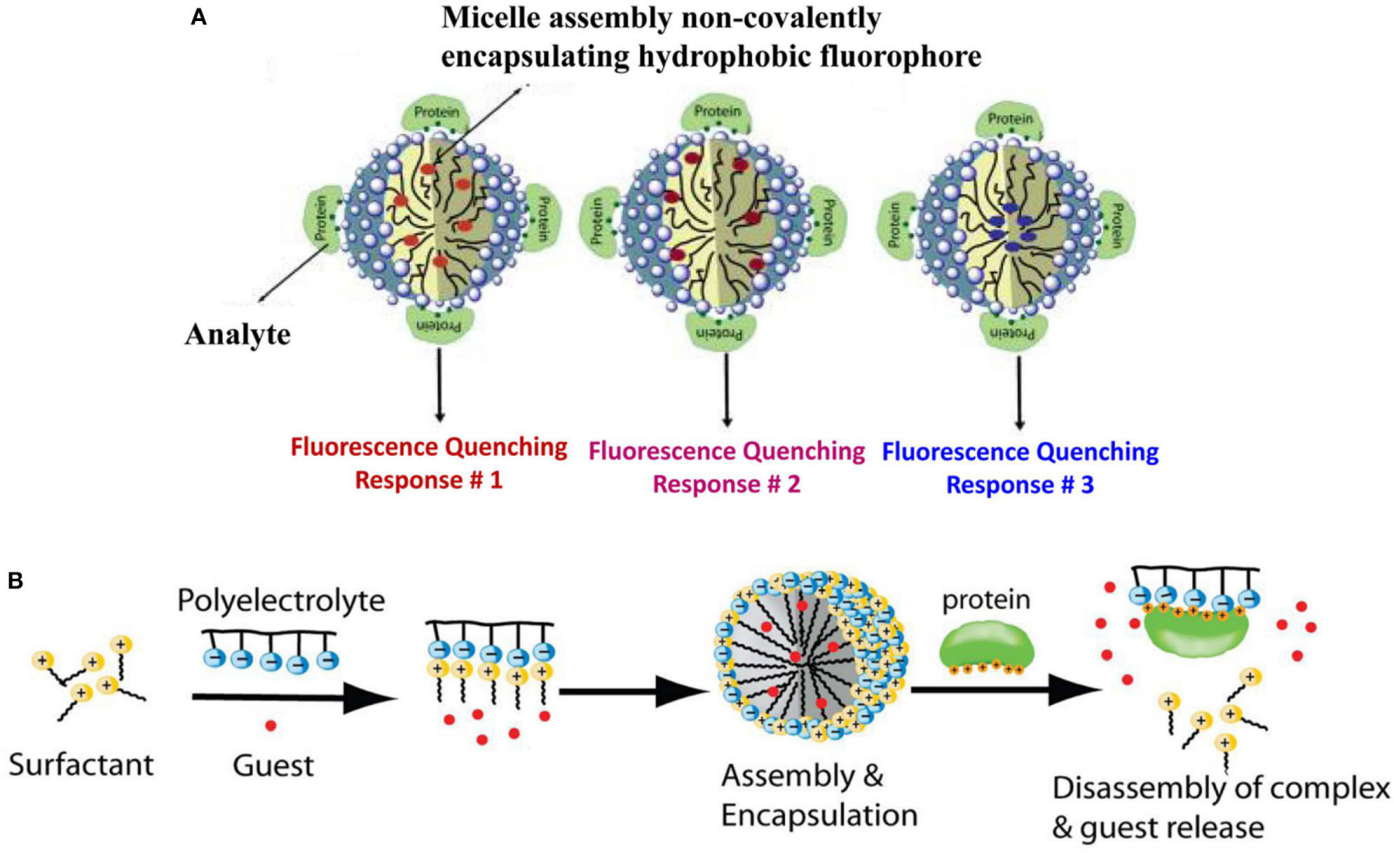
Schematic representation for protein recognition: **(A)** A single amphiphilic polymer encapsulated different dyes and **(B)** A surfactant-polyelectrolyte ensemble encapsulated different dyes. Reprinted from Sandanaraj et al. (2007) and Gonzalez et al. (2009) with permission.

Using this strategy, Thayumanavan et al. have developed simple methods for constructing sensor arrays for protein recognition by using one kind of amphiphilic aggregates to encapsulate different fluorophores (Sandanaraj et al., 2007; Gonzalez et al., 2009) or using different types of amphiphilic aggregates to encapsulate one fluorophore (Savariar et al., 2008). At first, they used a single amphiphilic polymer-based micellar host to encapsulate eight different hydrophobic dyes in its micellar interior and prepared an eight-element sensor array for distinguishing metalloproteins (Sandanaraj et al., 2007). Each ensemble showed different quenching responses to the four target metalloproteins and could generate a distinct recognition pattern by collecting the quenching signals (Figure 2A). Later, they fabricated a four-element sensor array using a negatively-charged surfactant and positively-charged polyelectrolyte formed micelle to encapsulate four dyes for discrimination of eight different proteins (Gonzalez et al., 2009). As shown in Figure 2B, protein-polyelectrolyte binding interaction could disassemble the supramolecular assembly, which released the guest dyes from the assembly interior. The different binding affinity between various proteins and polymer could produce distinct recognition patterns for protein discrimination. The main advantage of the present approach is that it is easy to construct a fluorescent sensor array by simple interchange of fluorophores, which spares time-consuming design and synthesis of multiple receptors. In addition, they also used different combinations of polyelectrolyte-surfactant assembly to encapsulate the same dye to build multiple-element sensor array for successfully distinguishing 5 proteins (Savariar et al., 2008). In 2018, Li et al. also demonstrated that the encapsulation of quantum dots-fluorophore FRET pair in different surfactant-polyelectrolyte nanomicelles could provide an efficient cross-reactive sensor for serum proteins (Li et al., 2018). Due to the completely non-covalent nature of the receptor component, the simplicity of the design makes this method highly versatile.

Choi et al. (2018) designed and synthesized four water-soluble aggregation-induced emission luminogens (AIEgens), which showed strong fluorescence upon addition of proteins by restricting the intramolecular rotation of AIEgens. The AIEgen-protein affinity was related to different chemical functional groups on AIEgens, which results in distinct fluorescence variations for each protein. The combined fluorescence outputs enabled accurate classification of five different proteins and specific proteins at different concentrations by LDA. This study provides a novel strategy for protein discrimination using amphiphilic fluorescent AIEgens as sensor elements.

### Quantum Dots

Quantum dots (QDs) are fluorescent materials which possess advantages like high fluorescence quantum yields, narrow and symmetric emission band, broad absorption, high resistance to photobleaching, large “effective” Stokes shifts, versatile surface modification, etc. (Boeneman et al., 2010; Tyrakowski and Snee, 2013; Zhang Z. et al., 2015) These unique properties make them useful for multiplexing in biological assay, such as long-term biological imaging (Jaiswal et al., 2003; Chen and Gerion, 2004), optical coding of cells (Gao et al., 2002; Mattheakis et al., 2004), diagnostic tools for cancer, etc. (Bakalova et al., 2004; Michalet et al., 2005; Mansur et al., 2014) Thus, QDs have emerged as attractive semiconductor materials in chemo/biosensing over the past few decades (Heuff et al., 2007).

**Figure 3:**
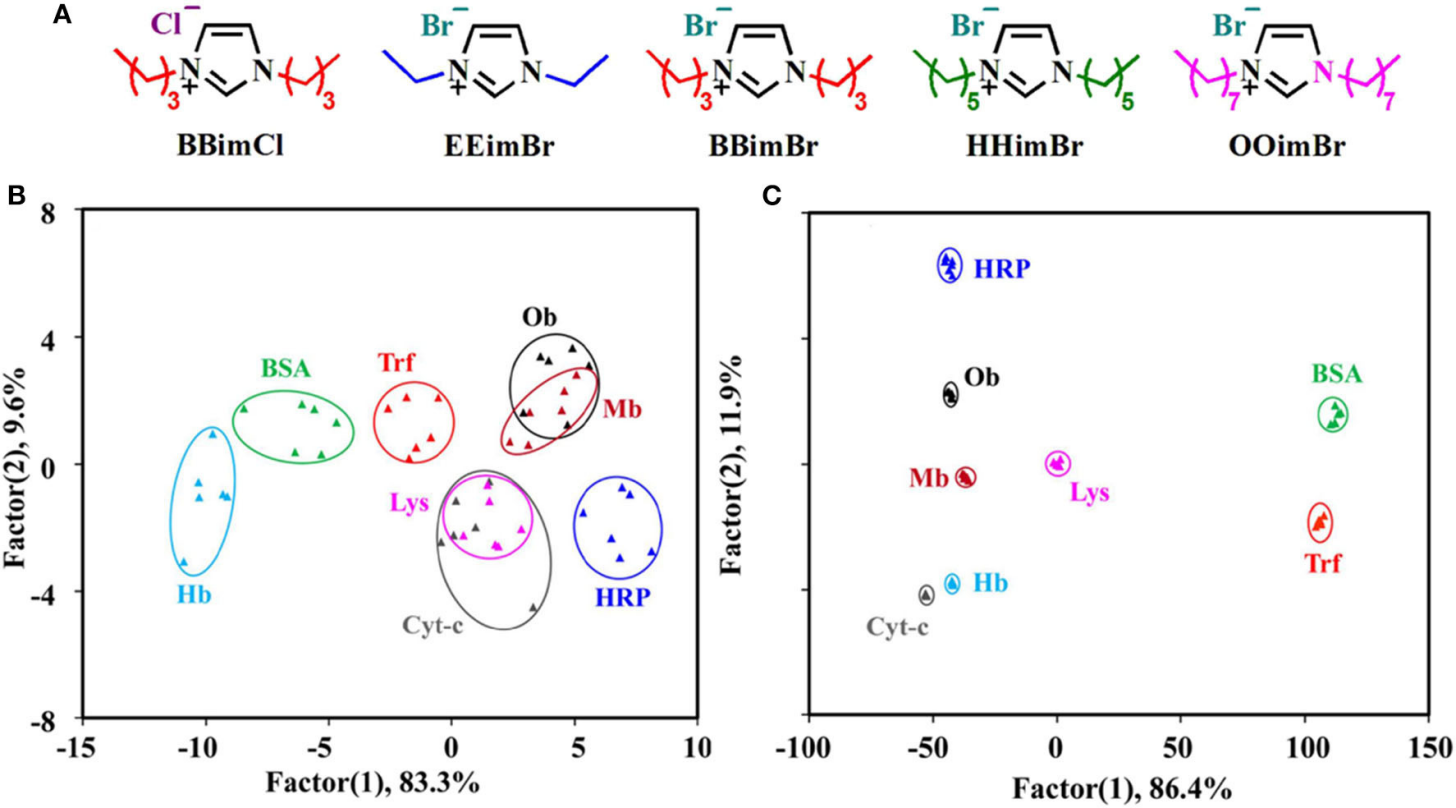
**(A)** Chemical Structures of five different ionic liquids. Canonical score plots for identifying eight proteins at 500 nM based on **(B)** ionic liquids and **(C)** ionic liquids-QDs. Reprinted from Chen et al. (2015) with permission.

QDs can form conjugates with ionic liquids or nanoparticles for constructing protein sensor arrays. Chen et al. (2015) reported that the introduction of QDs could improve the sensing sensitivity and discrimination accuracy of the ionic liquid-based sensor arrays due to the high quantum yields of QDs. They first constructed a sensor array containing five different ionic liquids (BBimCl, EEimBr, BBimBr, HHimBr, and OOimBr) and found it is difficult to distinguish 8 proteins at 500 nM completely (with an accuracy of 91.7%) (Figures 3A,B). Because the weak fluorescence of some ionic liquids limited the performance of this sensing system, they then employed CdTe quantum dots with strong fluorescence emission to form conjugates with ionic liquids and developed a new sensor array (BBimCl@CdTe, EEimBr@CdTe, BBimBr@CdTe, HHimBr@CdTe, and CdTe) to improve its recognition ability (Figure 3C). In this case, ionic liquids function as the main receptors to interact with proteins through hydrophobic and electrostatic interactions. CdTe QDs function as the main reporters and can also interact with proteins and lead to fluorescence variation because of the diminishing of surface defects. This sensor array can achieve the discrimination of proteins (LOD = 10 nM), protein mixtures, six physiologically relevant proteins spiked in human urine, and six strains of bacteria from three different species with 100% accuracy. Such an array system can be used as an easy-to-access, highly differentiated and adaptive tool for high-precision discrimination of biological targets. It also provides a novel method for the construction of sensitive sensor arrays for biological or diagnostic purposes.

Wang et al. (2017) developed a simple nanoparticle-quantum dot-based sensor array using six different nanoparticles and a single CdSe QDs, where nanoparticles serve as receptors and QDs as indicators. Fluorescence turn-on or further turn-off occurred because of the disruption between nanoparticles and QDs by proteins, creating distinct response patterns. LDA illustrated this array could well discriminate eight proteins and six cancer cells. This work indicates that the combination of nanoparticles and QDs offers arrays great potentials for medical diagnostics in future.

**Figure 4:**
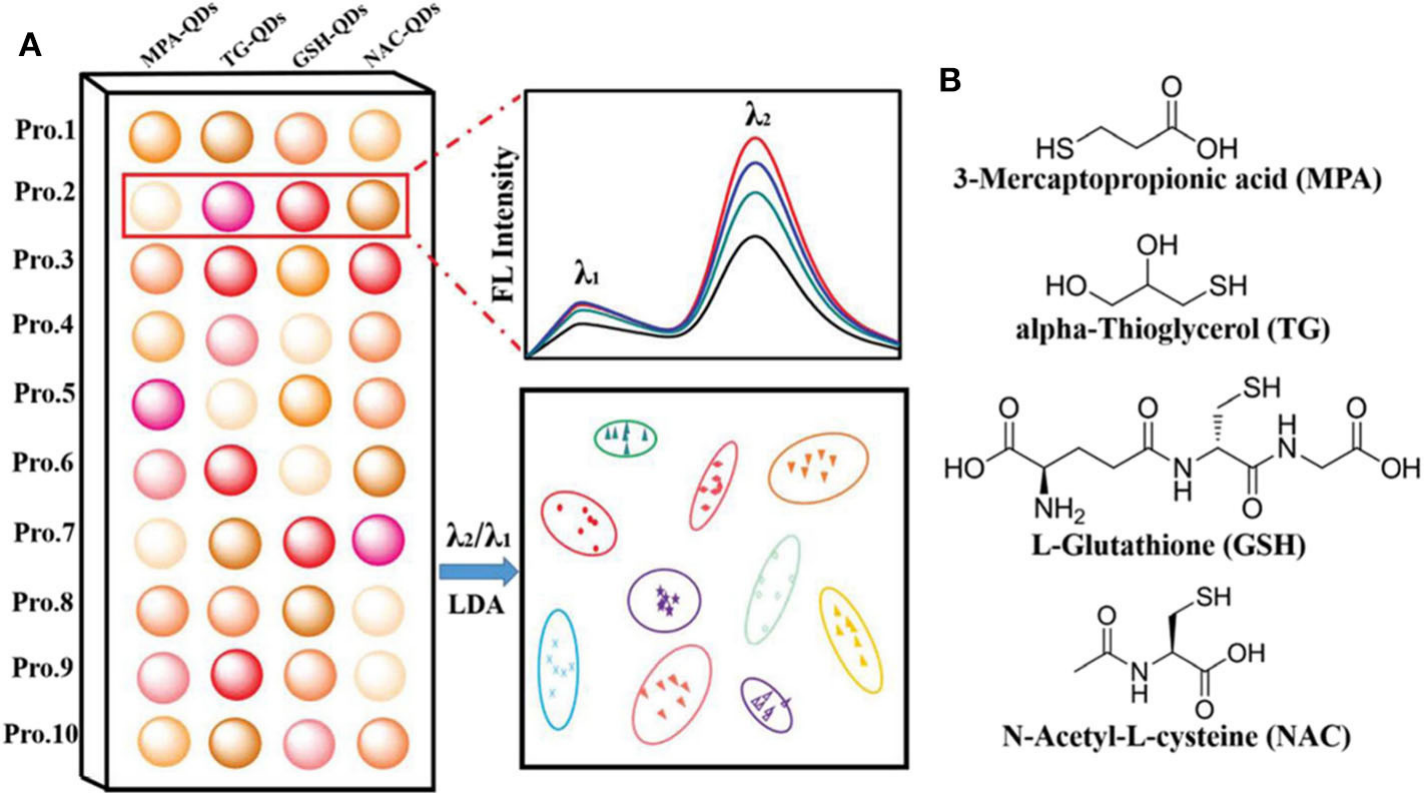
**(A)** Schematic illustration of the ratiometric sensor array based on various functionalized QDs for protein discrimination. **(B)** Chemical structures of the four ligands modified on the surface of QDs. Reprinted from Chang et al. (2016) with permission.

QDs can also be modified with different ligands to distinguish proteins. Chang et al. (2016) synthesized four dual-emitting Mn-doped ZnS QDs decorated with different functional ligands (MPA, TG, GSH, and NAC) as sensor elements for recognizing proteins (Figure 4). This ratiometric sensor array can discriminate pure proteins, unknown samples, and proteins in human urine with a high accuracy. Meanwhile, the authors found that the ratiometric signal can not only improve the recognition ability of sensor arrays, but also eliminate effectively the signal interference from pH changes. This research will open up new avenues to improve the discrimination capability of sensor arrays.

### Gold Nanoclusters

Gold nanoclusters (Au NCs) are composed of several to hundreds of gold atoms, which have gained great attention due to their ultra-small size, excellent biocompatibility and unusual photophysical properties (Lin et al., 2018; Xie et al., 2018). A large number of protected materials including polymers, proteins, peptides and DNA have been applied for preparing fluorescent Au NCs (Kong et al., 2012; Kwak et al., 2014; Zhang J. et al., 2015). And fluorophore-functionalized Au NCs have attracted much attention for protein recognition.

**Figure 5:**
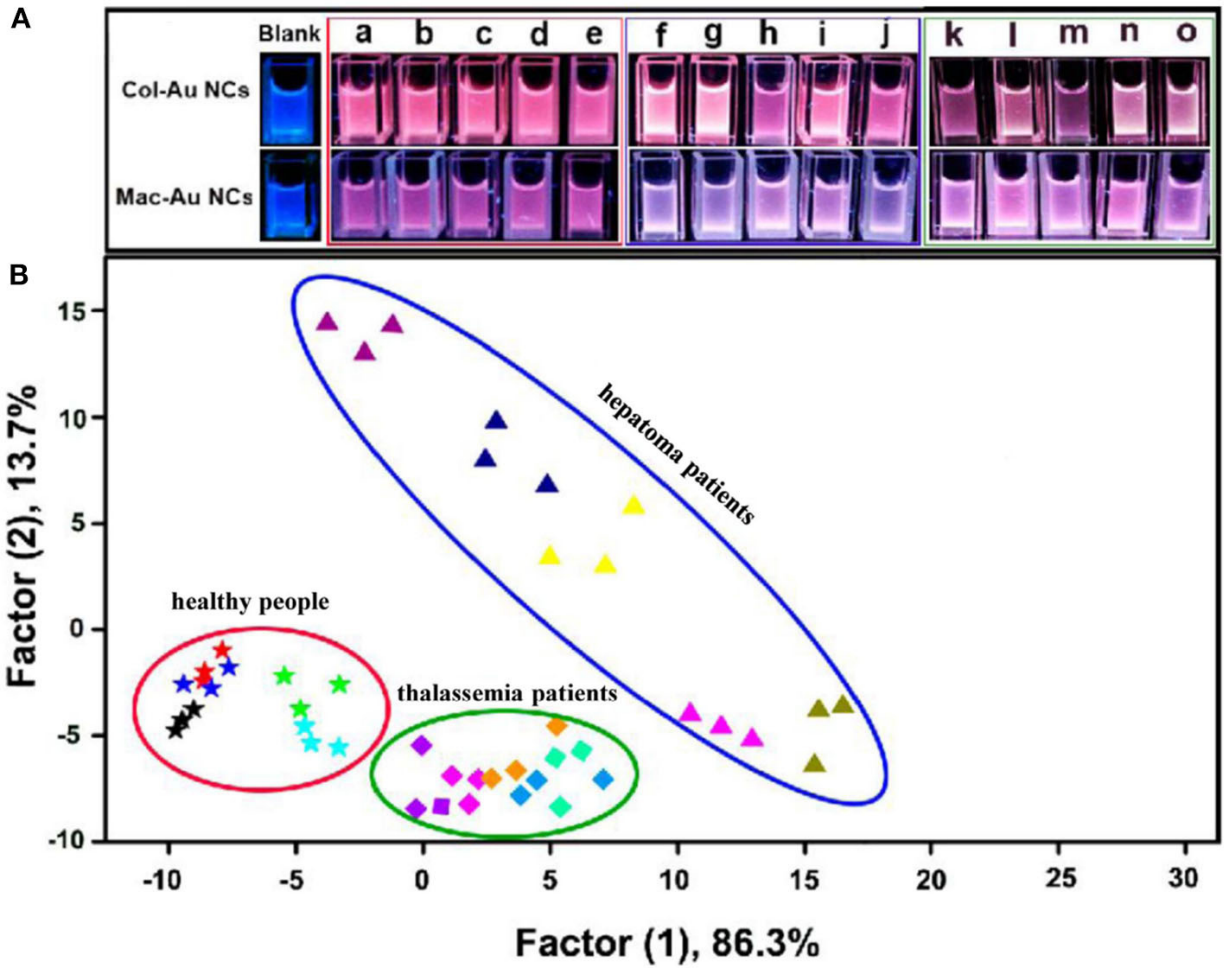
**(A)** Photographs of the Au NCs in the absence and presence of human serums from five normal people (a–e), five hepatoma patients (f–j), and five thalassemia patients (k–o) under UV lamp. **(B)** LDA score plot for discriminating different human serums. Reprinted from Xu et al. (2014) with permission.

Xu et al. (2014) developed a visual sensor array composed of two blue-emitting collagen or macerozyme R-10 protected Au NCs (Col-Au NCs and Mac-Au NCs) for pattern recognition of eight proteins. Experiment results illustrated that the possible mechanism was attributed to the protein-Au NCs complex formation. Furthermore, the present colorful array could effectively discriminate the serums from healthy people, hepatoma patients and thalassemia patients due to the different contents of certain proteins, showing potential application in clinical diagnosis (Figure 5). The same group also fabricated a sensor array based on five protein-protected Au NCs for protein discrimination, which was different from the traditional fluorescent sensor arrays due to the unexpected emission induced by luminol solution (Sun et al., 2017). This sensor array could identify seven proteins, because the added different proteins might have interactions with capping proteins on Au NCs, which further influenced the catalysis role of Au NCs on luminol emission. This research not only offers new insights of Au NCs into the emission of luminol, but also shows the potential for protein recognition.

In addition, Xu et al. (2017) built a protein sensor array based on six near infrared fluorescent amino acid-functionalized Au NCs containing two ligands, which have similar fluorescence profiles but different surface properties. These Au NCs could interact with proteins through hydrogen bonding and van der Waals forces, and the array could successfully distinguish ten proteins. More importantly, serums from three stages (early, middle, and late) of breast cancer patients and healthy individuals could also be identified, suggesting the potential applications in diagnosis of breast cancer.

### Gold Nanoparticles

Gold nanoparticles (AuNPs) have been extensively applied in biosensors because of their unique optical, catalytic, chemical and electronical properties, and these properties can be modulated by changing the shape, size, surface modification or aggregation state of AuNPs (Daniel and Astruc, 2004; Zhang et al., 2020). AuNPs are usually used to combine with DNA (Lu et al., 2013b; Sun et al., 2015), fluorophores (You et al., 2007; Bajaj et al., 2009; Rana et al., 2012), enzymes (Miranda et al., 2010), or surfactants (Rogowski et al., 2016; Xi et al., 2018) to construct non-specific protein sensor arrays.

### DNA-Gold Nanoparticle Conjugates

**Figure 6:**
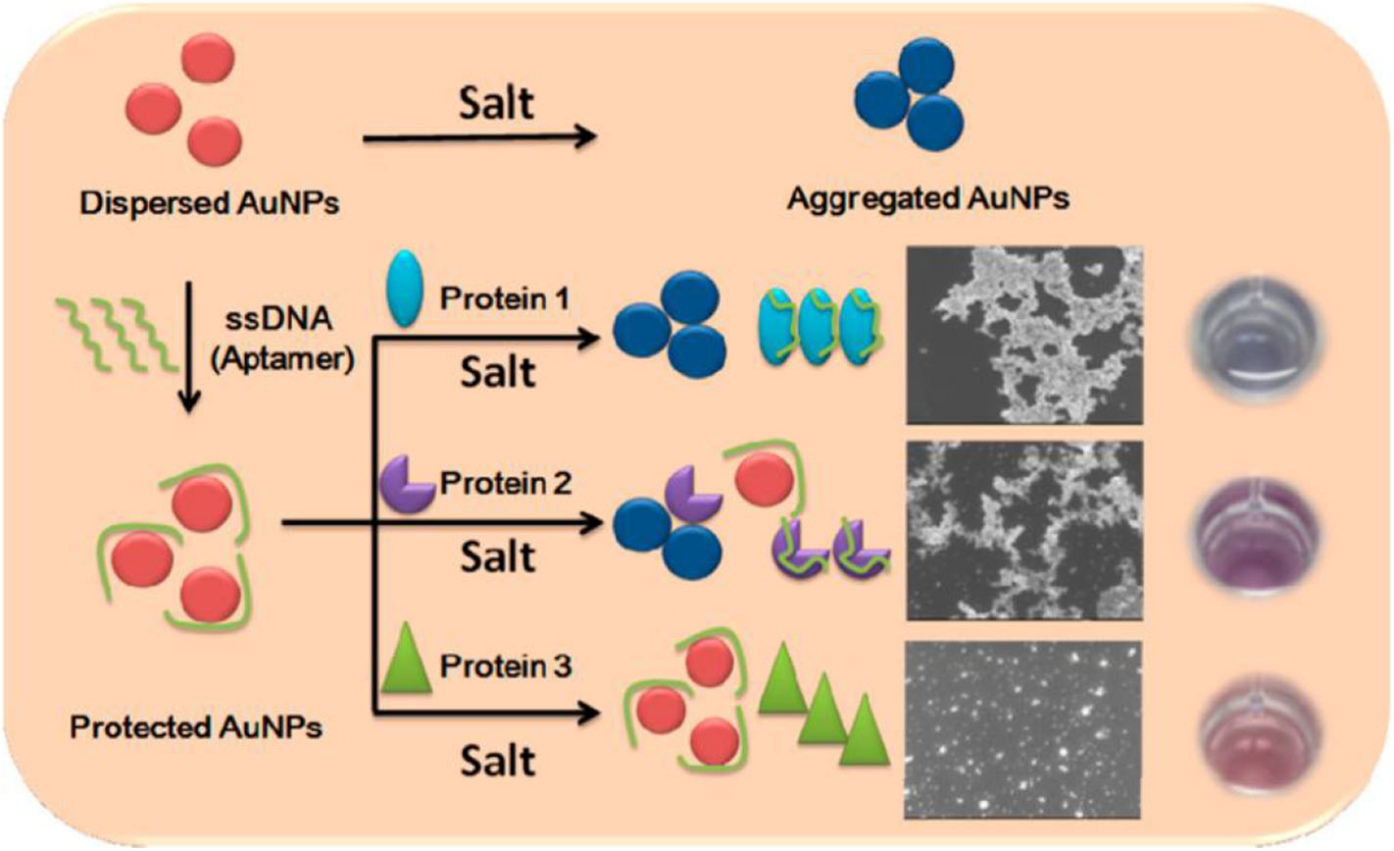
Schematic representation of the sensing principle of the aptamer-based sensor array. Reprinted from Lu et al. (2013b) with permission.

Researchers have built lots of DNA-AuNP conjugates-based arrays for protein identification due to the different interactions among proteins, DNA and AuNPs. Besides, DNA as a non-specific receptor could provide unlimited sensor elements for array sensing, because a short DNA sequence (e.g., 15 bases) has up to billions of combinations (Wei et al., 2017). In 2013, Lu et al. used three aptamer (DNA)-protected AuNPs as a colorimetric sensor array for protein discrimination (Lu et al., 2013b). They found that different proteins could make the DNA-AuNPs exhibit distinct aggregation behaviors in the presence of salt, causing different color change by naked eyes (Figure 6). LDA results illustrated that this sensor array could not only well distinguish seven proteins, but also identify human cancer cells and normal human cell with 100% accuracy. Similarly, Sun et al. (2015) built a protein sensor array containing three different DNA-AuNPs prepared by absorbing non-specific dye-labeled DNA sequences onto AuNPs. The added proteins could remove DNA from the surface of AuNPs because of the competitive binding, resulting in “turn-on” signals of the fluorescent dye and red-to-blue color changes due to the aggregation of AuNPs caused by salt. Both the fluorescent and colorimetric signals were used for identifying proteins, and this array could not only distinguish 11 proteins, but also discriminate bovine serum albumin (BSA) and human serum albumin (HSA) at different concentrations and the mixture with various ratios with an accuracy of 100% by LDA. Moreover, 10 proteins at the concentration of 1.0 μM could be well-discriminated in human urine.

Later, Wei et al. (2017) used three SH-labeled DNA functionalized catalytically active AuNPs as sensor elements for protein discrimination and found that the DNA-protein interactions could mask the surface of AuNPs, which further induced the poor catalytic performance of gold nanoparticles to the reaction between 4-nitrophenol and NaBH_4_. The color-change time of the reaction solution from yellow to colorless was taken as signal readout, and colorimetric response patterns could be obtained from this array. Eleven proteins even at 30 nM have been well-distinguished by LDA and naked eyes. Remarkably, the present array was able to completely identify among individual proteins and protein mixtures at different molar ratios. Besides, the practicability of this colorimetric array was demonstrated by discrimination of 11 proteins spiked in human serum with 100% accuracy.

In addition, Yang et al. (2013) developed a AuNPs-based colorimetric sensor array including five DNA-decorated catalytic AuNPs and one bare AuNPs for protein recognition. Each protein was first mixed with the sensing platform, and then HAuCl_4_ and NH_2_OH were added to make the nanoparticles grow. They selected three wavelengths related to the particle properties for each sensor element as absorbance data acquisition. This label-free 18-dimensional array (six sensor elements × three wavelengths) could effectively distinguish six different proteins, various concentrated proteins, protein mixtures and even samples in serum and urine. Later, they also used this type of sensor array to distinguish 4 different cell lines (Yang X. et al., 2014). In this type of sensor arrays, the addition of proteins can change the aggregation behaviors of DNA-AuNPs, remove DNA from AuNPs, or affect the catalytic performance of AuNPs. This protein detection method is simple, sensitive, and label-free, and it will provide new directions of developing array-based sensing systems and broaden the application field of nanoparticle-based sensors.

### Fluorophore/Enzyme-Gold Nanoparticle Conjugates

Rotello et al. have used different positively-charged AuNPs and one negatively-charged fluorescent polymer conjugate, green fluorescent protein (GFP), or enzyme to develop several fluorophore/enzyme displacement sensor arrays. In 2007, they developed a sensor array based on six different non-covalent cationic AuNPs and an anionic fluorescent polymer conjugate (You et al., 2007). The fluorescence of polymer conjugates was quenched by AuNPs, and this platform showed distinct turn-on response patterns to different proteins because the target proteins disrupted the interaction between AuNPs and polymer conjugates. This array could not only quantitatively differentiate seven proteins at nanomolar concentrations, but also identify 52 unknown protein samples with an identification accuracy of 94.2% by LDA. Using this strategy, they developed another array-based sensing system containing three AuNP-polymer conjugates to effectively differentiate normal, cancerous, and metastatic cells (Bajaj et al., 2009).

They also fabricated another effective sensor array containing five different positively-charged AuNPs and one negatively-charged GFP for protein recognition in buffer and human serum, where the fluorescence of GFP was quenched by AuNPs (De et al., 2009). The utilization of GFP instead of conjugated polymers minimizes the aggregation of sensor elements because GFP possesses lower aggregation than CPs, which enhances the quantum yield and sensitivity and then improves sensor efficiency. The addition of target serum proteins competed with GFP to bind AuNPs, leading to the fluorescence light-up or further quenching. It could discriminate 5 proteins at physiologically relevant concentrations in buffer (100% accuracy) and in human serum (97% accuracy). Furthermore, this array was also able to distinguish proteins at different concentrations, as well as the mixture of various proteins in human serum. This sensing strategy based on GFP-NP conjugates was further employed for rapidly identifying healthy and metastatic cancer cells and tissues, relying on the phenotypic differences of their overall proteome signatures (Bajaj et al., 2010; Rana et al., 2012). Notably, this eight-element sensor array created a distinct fingerprint for four normal and four metastatic tumor tissues and showed good discrimination ability. Overall, this sensing strategy provides the application prospect of unbiased phenotypic screening of tissue status caused by genetic variation and differentiation states.

In addition, they also constructed an enzyme-AuNP sensor array to improve the sensitivity through enzymatic catalysis (Miranda et al., 2010). In this approach, six AuNPs with positive surface charges were electrostatically bound to an anionic enzyme (β-galactosidase), inhibiting the activity of enzyme. The added target proteins released the enzyme, which restored activity and turned on the fluorescence of fluorogenic substrate. This signal-amplified sensor array was able to identify nine proteins at 1 nM in both buffer and desalted human urine.

The sensing mechanism of this type of sensor array is AuNPs could quench the fluorescence of polymer conjugates or GFP, or inhibit the activity of enzyme which turn off the fluorescence of fluorogenic substrate. Various proteins have different abilities to replace fluorophores/enzymes from AuNPs, thereby generating distinct turn-on signals, and then realizing the identification of proteins and cells. Using this strategy, the Rotello group has made outstanding contributions to the protein discrimination in human serum or urine and the identification of cells including healthy, cancerous, and metastatic cells.

### Surfactant-Gold Nanoparticle Conjugates

Colorimetric sensor arrays have been constructed for protein recognition by efficient surfactant-based AuNPs. Surfactants used in this strategy have the following advantages: (1) they can change the zeta potential of AuNPs; (2) they can act as protein receptors; and (3) they can adjust the protein-induced aggregation of AuNPs. Using a very simple washing procedure, Rogowski et al. (2016) prepared three stable AuNPs in cationic cetyltrimethylammonium bromide (CTAB), non-ionic Tween 20, and anionic sodium dodecyl sulfate (SDS) to fabricate a colorimetric sensor array for differentiating five proteins. The three types of surfactants not only changed the zeta potential of AuNPs, but also modulated the adsorption-driven aggregation of AuNPs by proteins. Moreover, the sensitivity could be altered by varying the surfactant concentration and the surfactant-coated AuNPs could be used to study the interactions between NPs and other analytes. Similarly, Xi et al. (2018) built a colorimetric sensor array for protein discrimination using cationic CTAB and two cationic polymers (chitosan and polydiallyl dimethylammonium chloride) as protein receptors and AuNPs as signal transducers. Positively charged polymers could bind to AuNPs in the absence of proteins, resulting in aggregation of AuNPs. The presence of negatively charged proteins caused different degrees of AuNP aggregation due to the diverse electrostatic interactions between proteins and polymers, giving rise to different signal variations. It could well differentiate seven negatively-charged proteins in Tris-HCl buffer and human serum samples. Such gold nanoparticle conjugates are easy to prepare because surfactants are commercially available. But so far, this type of sensor array is limited to colorimetric sensor arrays.

### Other Nanoparticles

**Figure 7:**
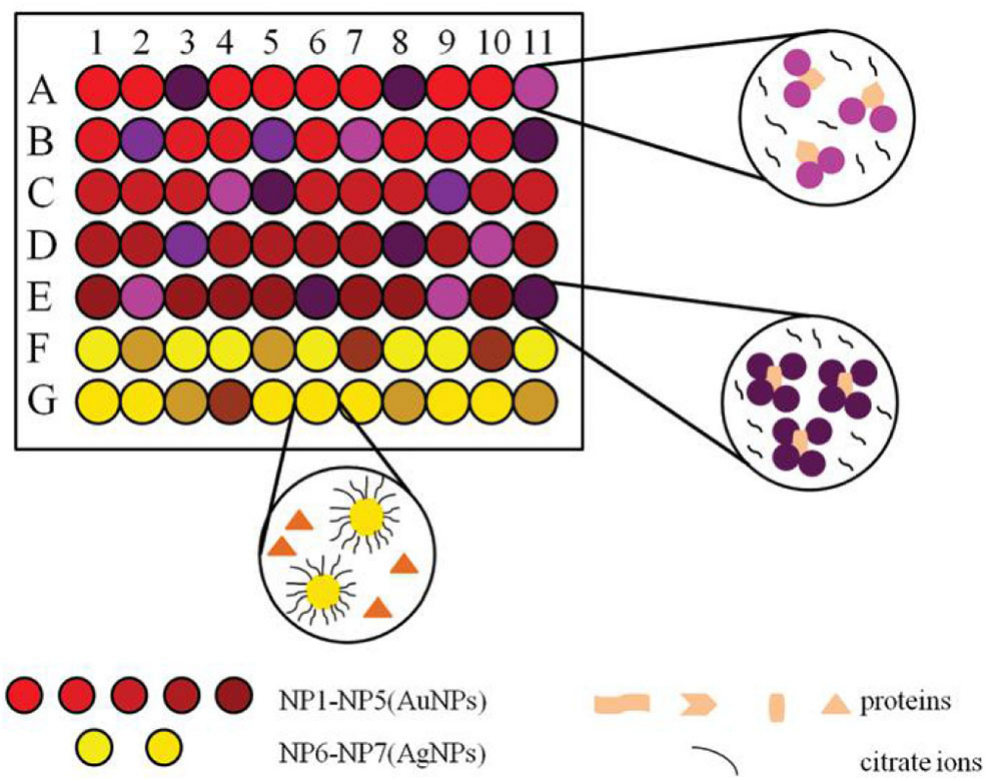
Schematic representation of a sensor array based on seven noble metal nanoparticles for protein recognition. Reprinted from Zhang S. et al. (2015) with permission.

The unmodified noble metal nanoparticles, other metal nanoparticles and nanodots are also receiving extensive attention in the field of protein sensing. Zhang S. et al. (2015) fabricated a colorimetric sensor array utilizing seven unmodified noble metal nanoparticles (2 AgNPs and 5 AuNPs) with different sizes (Figure 7). The absorbance of the nanoparticles changed differently in the presence of ten proteins, which produced distinct response patterns visually distinguished by naked eyes. These proteins at different concentrations could be further successfully identified by LDA. Moreover, this array was able to discriminate seven bacteria and four cancer cells correctly. This assay illustrated that the sensor array based on unmodified noble metal nanoparticles has application potential in medical diagnostics.

Kong et al. (2011) constructed a sensitive and effective colorimetric sensor array based on catalytically active Fe_3_O_4_ NPs for protein discrimination. By altering the surface functionality, the two-element array (dopamine-Fe_3_O_4_ NPs and trimethylammonium-Fe_3_O_4_ NPs) was able to identify 10 proteins, and differentiate unknown samples with an accuracy of 95%. Nandu et al. (2018) reported a five-element sensor array containing two-dimensional nanoparticles (2D-nps, including MoS_2_, WS_2_, and nGO) and fluorescently labeled ssDNA after a careful screening/filtering process. The displacement of ssDNA from the surface of 2D-nps made the array able to discriminate seven proteins.

Tao et al. (2014) used seven luminescent nanodots and graphene oxide (GO) to create a protein sensor array. The fluorescence of these nanodots was quenched by GO, and the presence of various proteins disrupted the nanodot-GO interaction to different extents. LDA was applied to successfully identify 10 proteins at nanomolar concentrations and discriminate 50 unknown samples with 94% accuracy. Furthermore, this array could be expanded to distinguish non-resistant and drug-resistant bacteria which express different proteins, endowing the potential applications in diagnosis of bacterial infections. Besides, Yuan et al. (2015) synthesized eight dual-ligand co-functionalized gold nanodots (Au NDs) with diverse surface and similar fluorescence properties to fabricate a sensor array for proteins, which could well discriminate eight proteins and 48 unknown protein samples (100% accuracy). Clearly, nanoparticle-based sensor arrays are not limited to gold nanoparticles.

To construct a successful array-based optical sensing system, analytes and cross-reactive sensor elements should possess different interactions that lead to distinct responses. Multi-element-based sensor arrays for proteins have been widely developed and the detection mechanism, advantages and disadvantages for different types of arrays are summarized and analyzed in Table 1.

**Table 1:** Summary of different types of multi-element-based sensor arrays.

**Type**	**Detection mechanism**	**Advantages**	**Disadvantages**
Conjugated polymers	The formation of protein-CP complexes causes aggregation/size changes of CPs.	The direct interaction between CPs and proteins makes the array very sensitive.	The synthesis and purification of CPs is complicated.
Amphiphilic aggregates	Non-metalloprotein-binding induces the amphiphilic ensemble disassembly or assembly, and energy/electron transfer occurs from the probe to metalloproteins.	It is easy to fabricate such arrays by changing the encapsulated fluorophores or amphiphilic aggregates.	The amphiphilic molecule used needs to be charged and there are few fluorescent amphiphilic aggregates for protein recognition.
Quantum dots	The added proteins can disrupt the interaction between QDs and ionic liquids or QDs and nanoparticles.	It can improve the sensing sensitivity and discrimination ability of arrays due to their high quantum yields.	The interaction between QDs and proteins is not clear, which limits the construction of arrays that only use QDs for protein identification.
Gold nanoclusters	Protein-Au NCs complex is formed.	This type of array has ultra-small size, excellent biocompatibility, etc.	Gold is a precious metal, which makes the preparation of Au NCs expensive.
Gold nanoparticles	Proteins disrupt the interaction between DNA and AuNPs, or fluorophore/enzyme and NPs, or surfactant and AuNPs.	The optical properties could be well-modulated by changing the size, shape, surface modification or aggregation state of the AuNPs.	Gold is a high-cost metal and the construction of such arrays is complex due to the introduction of DNA fluorophores, or surfactants.

## Environment-Sensitive Sensor Arrays for Protein Recognition

Environment-sensitive sensors are a type of sensors that are dependent on the physical and chemical properties of the surrounding environment. It is relatively easy to fabricate an environment-sensitive sensor array because it can be achieved by adjusting polarity, viscosity, pH, etc. (Vazquez et al., 2005; Yang Z. et al., 2014; Daly et al., 2017) Discriminative optical sensor arrays for proteins were also constructed by changing the pH values and ionic strengths because different proteins usually have different net surface charges, such as negative, positive, or neutral at various pH. Xu et al. (2010) created an array of water-soluble conjugated polymer solutions with six different ionic strengths for protein identification and denaturation detection. The conjugated polymer could form complexes with proteins *via* electrostatic or hydrophobic interactions, which exhibited different fluorescent responses. Results suggested that the ionic strength of testing solution could affect the electrostatic interaction and protein aggregation. Thus, this sensor array could identify seven different proteins and also detect denaturation of proteins.

**Figure 8:**
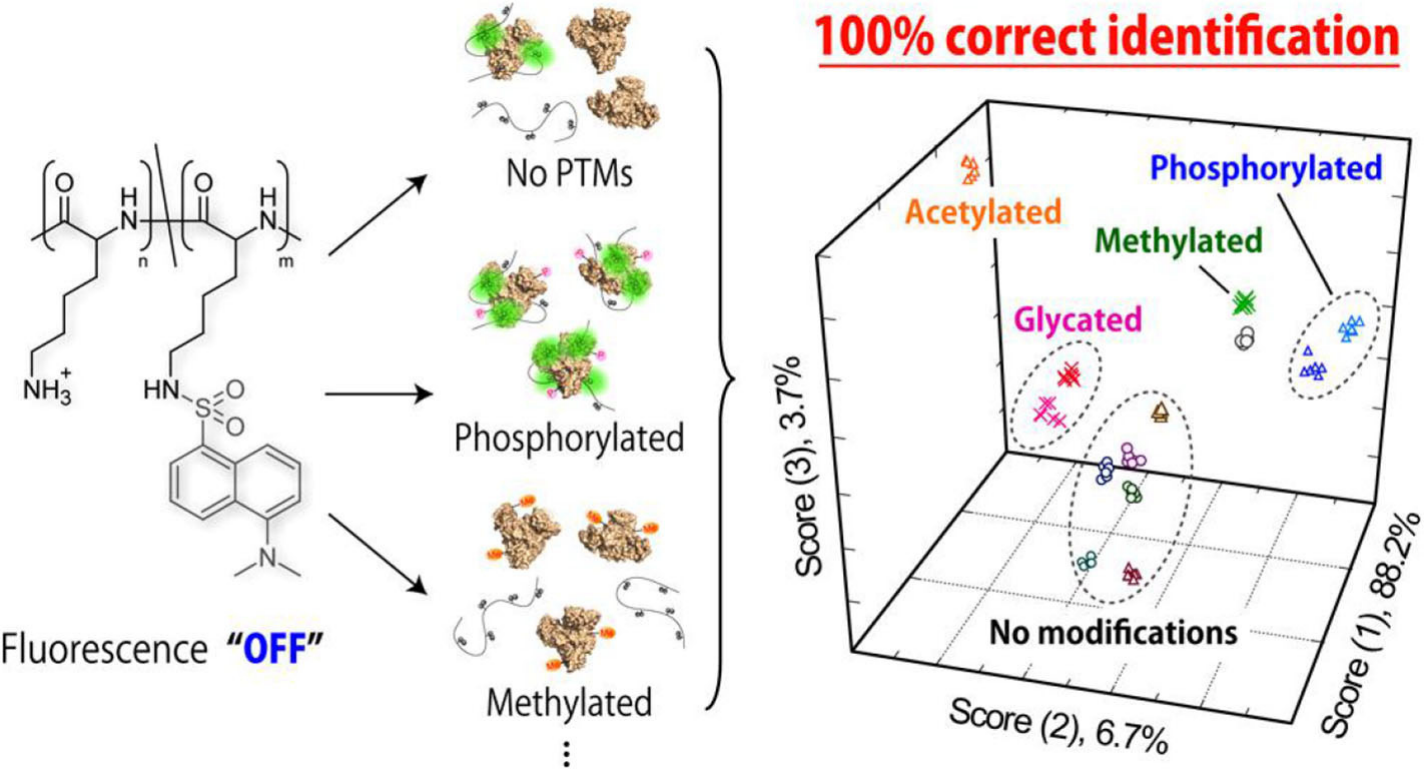
Schematic illustration of fluorogenic interactions with proteins with/without PTMs. Reprinted from Tomita et al. (2017) with permission.

The discrimination of post-translational modifications (PTMs) in proteins has attracted widespread attention in the elucidation of human diseases as well as therapeutic protein improvements (Venne et al., 2014; Pagel et al., 2015; Dumont et al., 2016). Tomita et al. (2017) reported a novel strategy based on a poly-L-lysine (PLL-Dnc) derivative modified with environment-sensitive dansyl fluorophore for fingerprinting protein populations with PTMs. They used this single PLL-Dnc polymer at six different pH values and ionic strengths to construct a sensor array for successfully distinguishing proteins (Figure 8). It was not only capable of identifying four mammalian serum albumins (HSA, BSA, ESA, and RSA), but also separating the protein populations with/without PTMs (phosphorylated, glycated, methylated, and acetylated proteins). This is the first work for applying a single synthetic polymer to discriminate multiple proteins with/without PTMs. In order to promote the application of this system in detecting proteins secreted by cultured cells, the same group studied the ability of PLL-Dnc to recognize hepatocyte-derived secreted proteins incorporated into culture media (Sugai et al., 2019). An array of PLL-Dnc in six different buffer solutions could successfully recognize five secretory proteins (α_1_-antitrypsin, albumin, transferrin, fibrinogen, and α-fetoprotein) in culture media with 100% accuracy. This work proved the solution-condition-dependent discrimination of proteins spiked into the culture medium, rendering such an environment-sensitive system a potential sensor platform for evaluation of cultured cells.

In addition, pH-based sensor arrays using QDs or Au NCs were also developed. Yan et al. (2019) reported a sensor array containing negatively charged QDs as an indicator for protein discrimination in different pH buffer solutions. The results illustrated that proteins with different isoelectric points (pI < 7, pI = 7, or pI > 7) could be differentiated successfully. It is known that pI is defined as the pH value at which a protein has no net charge. A protein possesses net negative (positive) surface charge when pH is above (below) its pI, and thus has different electrostatic interactions with QDs, which contributes to the differentiation of different proteins. Furthermore, this sensor array was able to identify complex protein mixtures and HSA at different concentrations in water and urine. Subsequently, Xu et al. (2019) synthesized a type of Au NCs coated with screened peptides of specific sequences, and developed a sensor array with different positive or negative charges through adjusting pH values (pH 1.5, 3.5, 6.0, 10.0, and 11.5). This sensor array could identify not only 10 proteins, but also protein mixtures with diverse components. Furthermore, a total of 50 unknown proteins could be effectively discriminated in human urine with 100% accuracy, and serums from rectal cancer patients, severe osteoarthritis patients, breast cancer patients and healthy people could be well-identified, suggesting possible potential application in auxiliary diagnosis. So far, the environment-sensitive sensor arrays for proteins have been constructed only by adjusting pH values and ionic strengths. Therefore, it is urgent to develop novel types of environmental-sensitive sensor array, for example, by changing the viscosity, polarity, etc.

## Multi-Wavelength-Based Single Sensing System for Protein Recognition

The key to develop multi-wavelength-based single system is to provide diverse signal variations at different wavelengths, where this system can generate distinct response patterns to various analytes at an array of channels (Lu et al., 2013a; Sang and Wang, 2014; Long et al., 2020) or emission bands (Kstereli et al., 2014; Hatai et al., 2017). Thus, this kind of sensing system can be divided into multi-channel sensors and multi-band emission sensors.

### Multi-Channel Sensors

Multi-channel sensors have attracted much more attention because they can extract multidimensional signals from an individual multifunctional sensor element, which is called “lab-on-a-molecule” or “lab-on-a-nanoparticle” (Wu et al., 2011; Yang B. et al., 2014). Multiple signal transduction, such as UV-vis absorbance, fluorescence (FL), phosphorescence (Ph), electrogenerated chemiluminescence (ECL), light scattering (LS), electrochemical (EC), turbidity and so forth, have been involved into nanoparticles or graphene oxide for multi-channel sensing (Liu et al., 2010; Hu et al., 2011; Wu et al., 2013). Up to now, a lot of dual-channel, triple-channel, and quadruple-channel sensors have been widely developed for protein identification.

**Figure 9:**
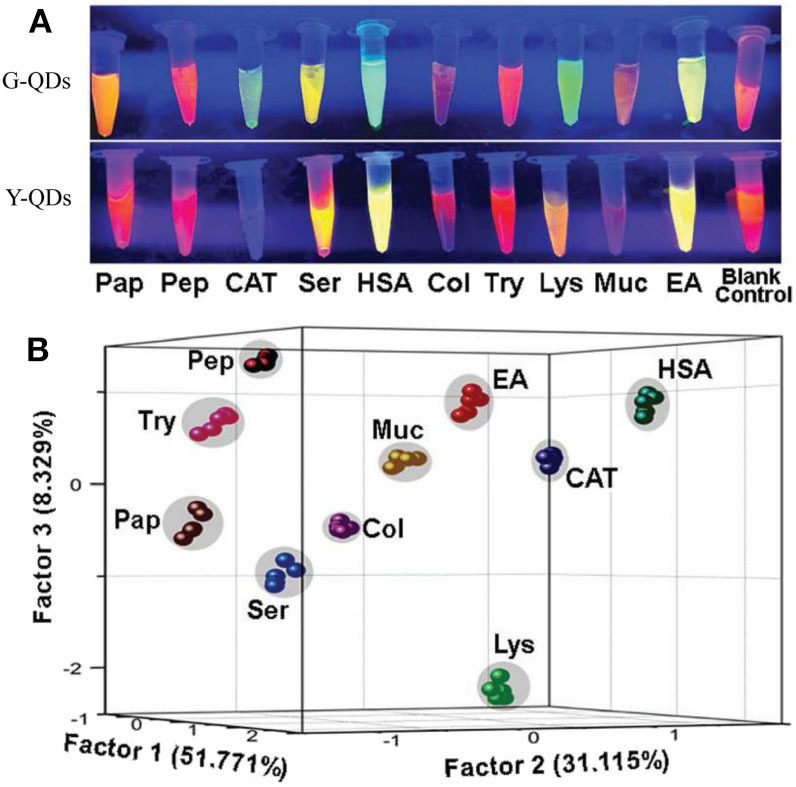
**(A)** FL variation images of G-QDs and Y-QDs with and without proteins under the UV-light irradiation. **(B)** PCA canonical score plot for identifying corresponding proteins. Reprinted from Ma et al. (2017) with permission.

For dual-channel sensors, Ma et al. (2017) have established a fluorescent sensor based on light-induced self-assembly behavior of bi-color thioglycolic acid-capped CdTe QDs (green and yellow emitting QDs, i.e., G-QDs and Y-QDs), which were prepared by simply modulating the reaction time (90 and 105 min). The added proteins and CdTe QDs could form CdTe-protein complex which would further influence the self-assembly behavior of CdTe QDs and result in diverse changes of FL signals (Figure 9). This dual-channel sensor was successfully applied for discrimination of 10 native proteins, 10 thermally denatured proteins, and 8 native proteins in urine. This study has provided a simple and visual method for protein identification, which may be further applied in studying the conformational changes of biomacromolecules.

For triple-channel sensors, they are very popular and have been largely used to construct protein sensors based on different materials. Lu et al. (2013a) found that graphene oxide could interact with proteins and affect its fluorescence, catalytic activity and assembly behavior. Thus, a triple-channel optical sensor (fluorescence, colorimetric and turbidity) was proposed for protein discrimination in a “lab-on-graphene” manner. Six different proteins, unknown samples and protein mixtures were all well-distinguished by LDA with 100% accuracy, respectively. Considering the good biocompatibility and variety of graphene-based materials, this study will broaden the potential application fields of graphene-based materials.

**Figure 10:**
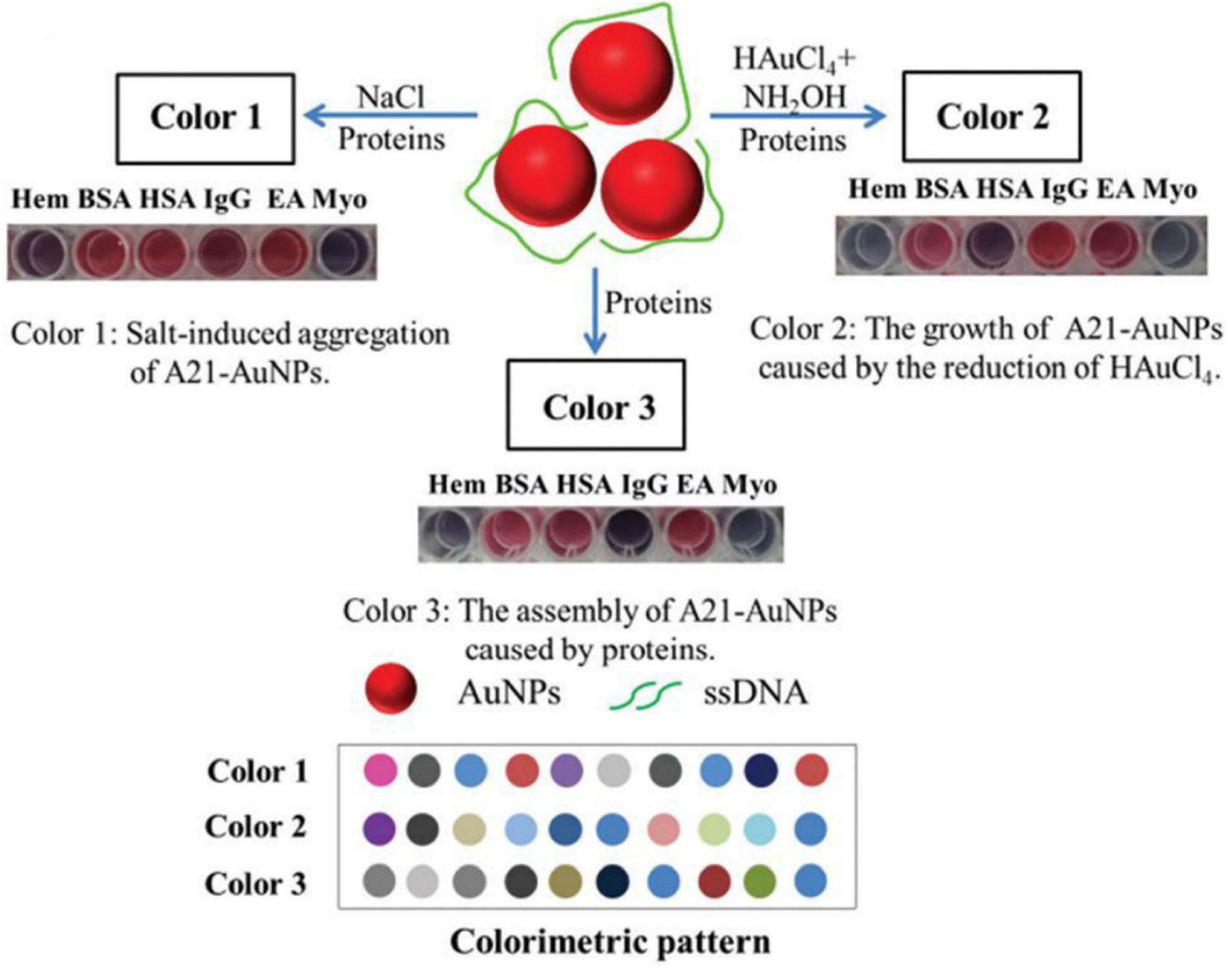
Schematic representation of the triple-channel A21-AuNPs for protein sensing and the produced colorimetric pattern. Reprinted from Mao et al. (2016) with permission.

The Liu group fabricated a triple-channel colorimetric sensor based on DNA-AuNP conjugates (A21-AuNPs) for protein discrimination (Mao et al., 2016). The triple-channel signals are composed of salt-induced aggregation (color 1), HAuCl_4_-induced AuNP regrowth (color 2) and protein-triggered aggregation (color 3) (Figure 10). Different proteins have diverse degrees of disruption of the interaction between DNA and AuNPs, which could make the response signals change differently. Thus, such a sensor was able to effectively identify 13 proteins, BSA and HSA mixtures, unknown samples and proteins in urine with an accuracy of 100%. They also developed another triple-channel colorimetric sensor by collecting the absorbance signals at three different reaction time points (10, 15, and 20 min) (Yang et al., 2017). Ten proteins in both aqueous solution and human urine were discriminated with 100% accuracy. Moreover, this optosensing system could quantitatively detect HSA and identify mixtures of HSA and Lys at different molar ratios in urine. This assay illustrated the use of real-time resolved response signals could increase the recognition ability for protein discrimination.

QDs are also applied for constructing multi-channel sensors by collecting the signal changes of one type of QDs at different channels. Wu et al. (2011) reported a triple-channel sensing device based on the optical intensities of FL, LS, and Ph of Mn-ZnS QDs for protein discrimination. Distinct recognition patterns related to eight proteins can be generated by collecting the three response signals of this triple-channel sensor. This sensor was further applied for identification of proteins in human urine to demonstrate its potential application. Sang and Wang (2014) also constructed a triple-channel sensor based on Mn-ZnS QDs for 10 glycoprotein identification by utilizing the fluorescence polarization (FP), Ph, and LS signals. The tested 10 glycoproteins could be well-distinguished in PBS buffer and human serum. Besides, the accuracy of identifying unknown samples was above 96%.

**Figure 11:**
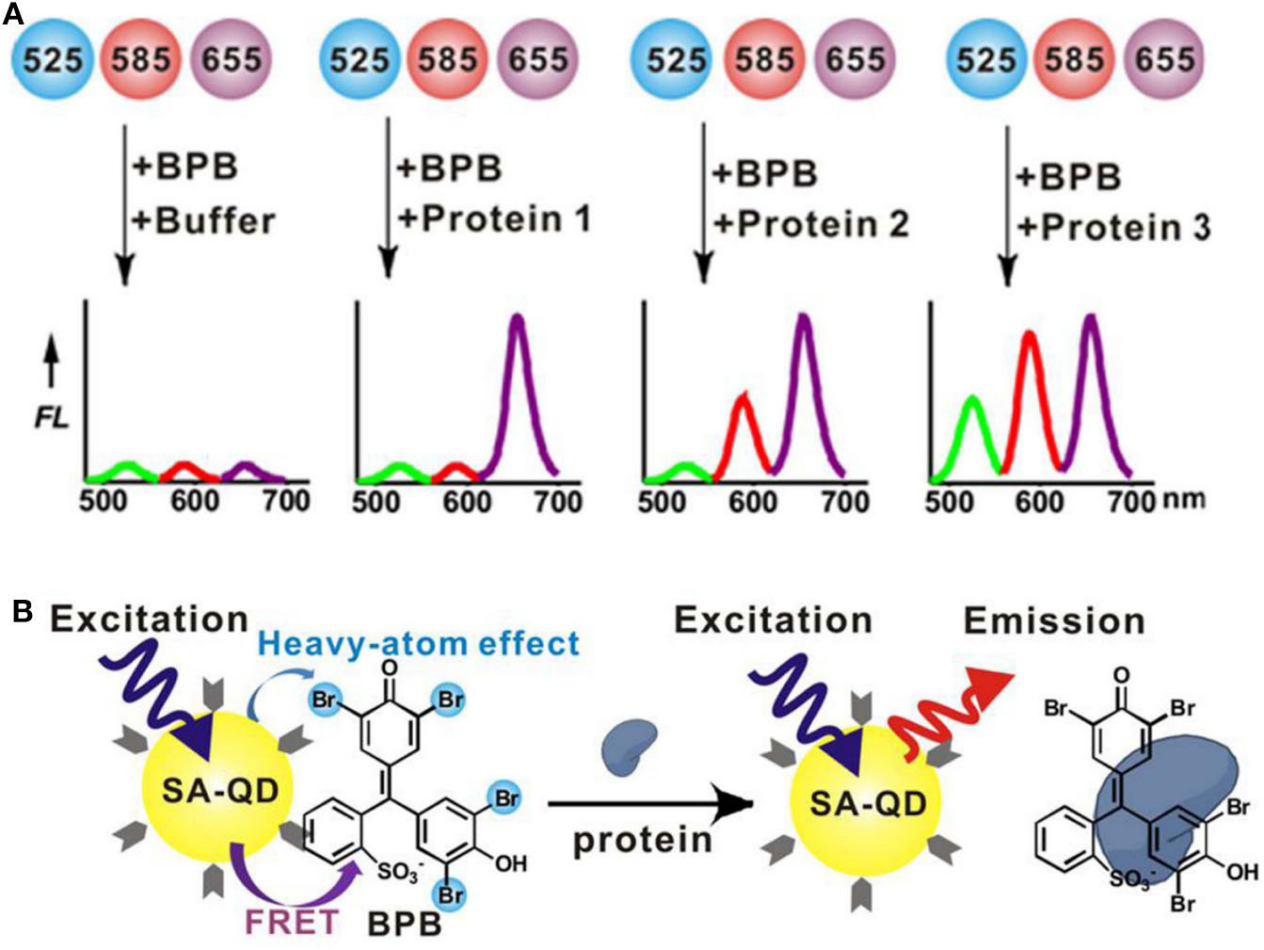
**(A)** Workflow of a triple-channel sensor for differentiation of proteins. **(B)** Schematic illustration of protein binding-induced displacement of BPB. Reprinted from Xu et al. (2016) with permission.

Then, Xu et al. (2016) have built a triple-channel sensor containing three spectral resolvable streptavidin (SA)-QDs (QD525, QD585, and QD655) and a quencher (bromophenol blue, BPB) in a single solution (Figure 11A). The quencher BPB could non-covalently bind with QDs to form QD-BPB complex, inducing fluorescence quenching of QDs. The addition of proteins disrupted the QD-BPB complex, leading to the separation of BPB from QDs and the fluorescence recovery of QDs (Figure 11B). This sensing platform excited at a single-wavelength allows for the correct identification of 10 proteins with very rapid response time (within 1 min). Moreover, it can apply in discrimination of proteins in serum and recognition of seven cell lines (one normal human cell line and six cancerous cell lines) based on the different cell surface proteins, suggesting great potential for fast and high-throughput medical diagnostics.

**Figure 12:**
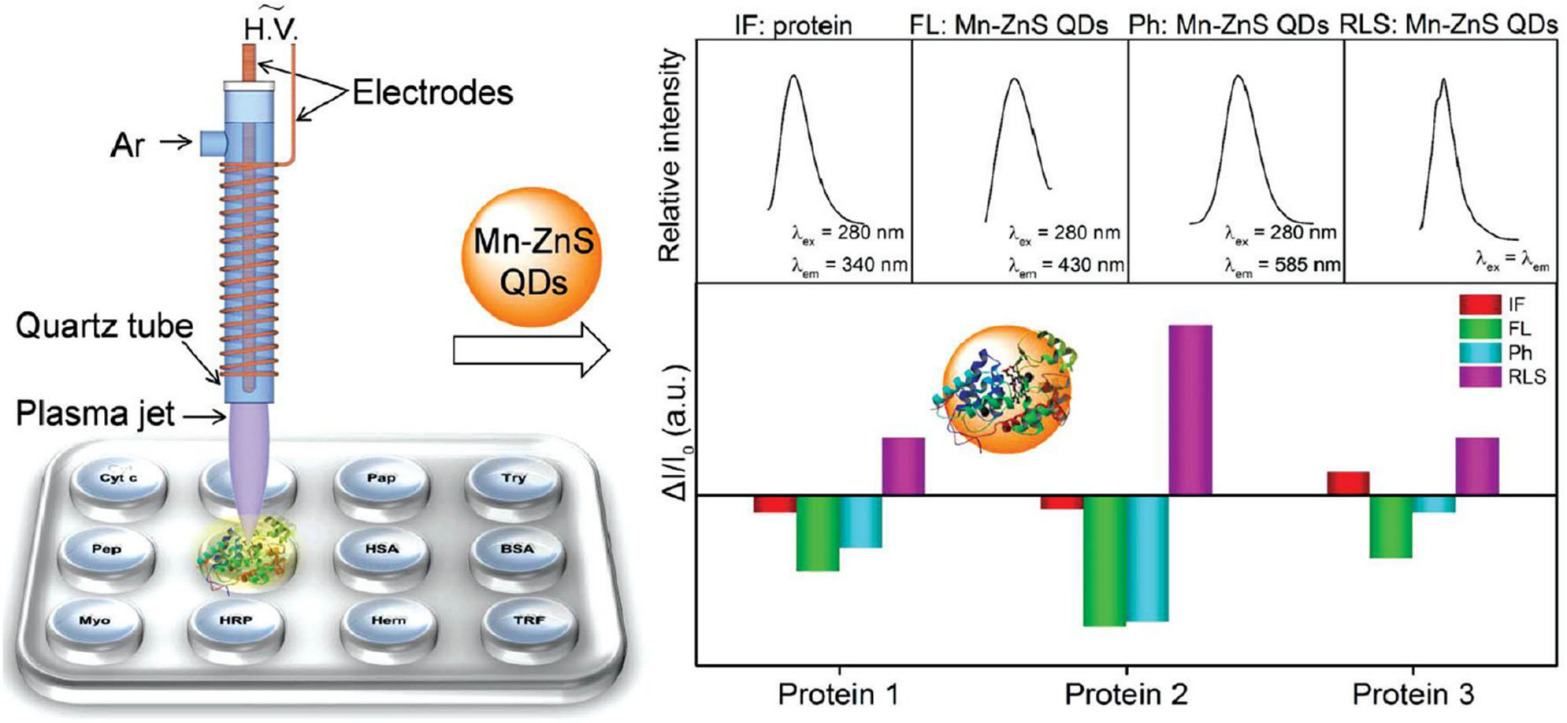
Schematic representation of the plasma-assisted quadruple-channel sensing device for protein discrimination. Reprinted from Li et al. (2016) with permission.

For quadruple-channel sensors, Li et al. (2016) constructed a sensing device including the intrinsic fluorescence (IF) of proteins and the triple-channel optical output of Mn-ZnS QDs (FL, Ph, and LS) (Figure 12). They found that the introduction of IF as the fourth channel could dramatically improve the protein discrimination resolution. To further enhance the cross-reactivity of the sensor, they applied dielectric barrier discharge plasma for protein modification to increase the IF difference of proteins as well as interactions between proteins and QDs. This sensor could precisely distinguish 12 diverse proteins, mixed serum proteins, and physiologically relevant proteins in human serum and urine with 100% accuracy. Furthermore, it could discriminate three different kinds of cell lines including human normal cell, cancerous cell, and mouse metastatic cell. The developed multi-channel sensors are mainly based on QDs, AuNPs, and graphene oxide. In contrast, the development of triple-channel sensors is relatively rapid. The challenge of this type of sensor is to find new materials and develop low-channel sensors.

### Multi-Band Emission Sensors

Multi-band emission sensors are sensors that perform discriminative sensing based on signal changes at different emission wavelengths. The Margulies group put forward the concept of combinatorial fluorescent molecular sensor which was obtained by introducing several fluorescent units with various emission bands and different recognition units into the same molecular structure. The binding of different analytes caused distinct changes at different wavelengths, consequently resulting in distinguishable signatures (Rout et al., 2012, 2013; Sarker et al., 2016). In 2017, they designed and synthesized a unimolecular fluorescent probe containing four partially spectrally overlapping fluorescent dyes (nitrobenzoxadiazole, Nile red, cyanine 5.5, and cyanine 7) and three specific binders for different protein families (Pode et al., 2017). This probe can overcome the shortcomings of some current probes that are less suitable for analyzing specific protein populations in their natural environment. It could not only identify different proteins, but also discriminate combinations of protein families in complex mixtures. More importantly, it could distinguish among isoforms in living cells that are inaccessible to macroscopic arrays.

**Figure 13:**
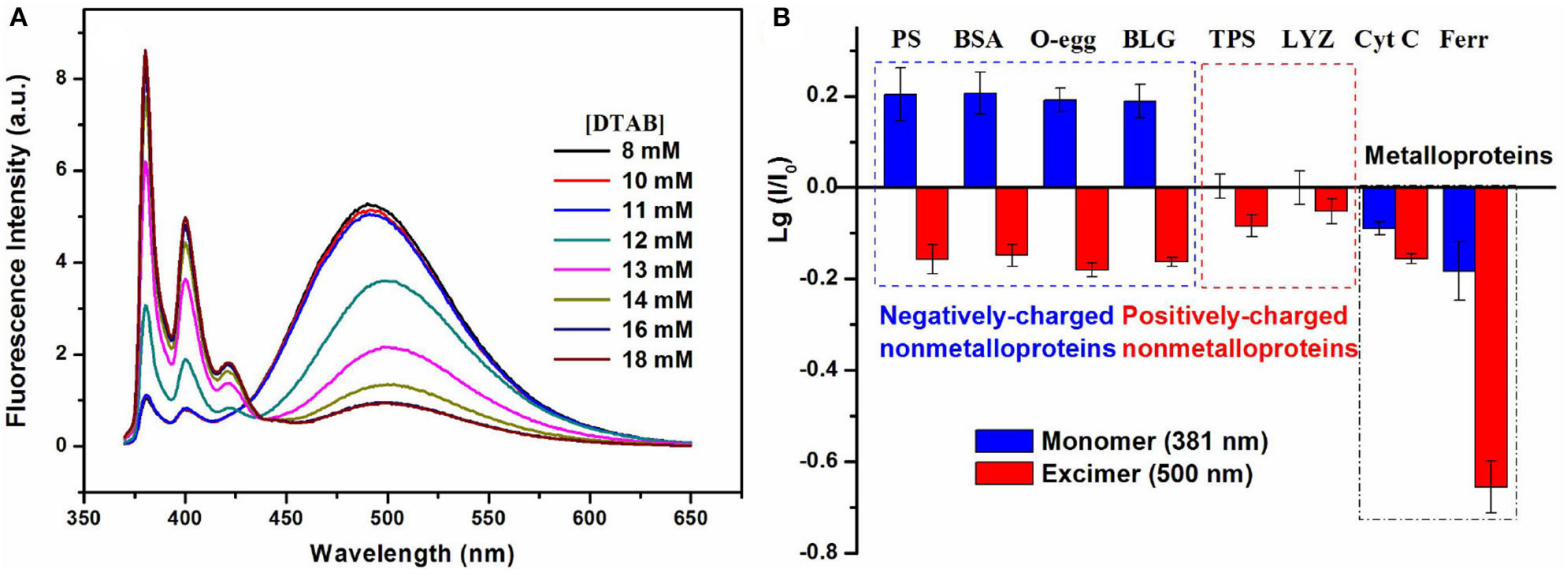
**(A)** Fluorescence emission spectra of the bispyrene probe in different concentrated DTAB solutions. **(B)** Recognition patterns for different types of proteins. Reprinted from Fan et al. (2015) with permission.

Using the surfactant encapsulating and modulating effect, our group has fabricated a series of multi-wavelength-based cross-reactive sensors for protein identification by designing a fluorescent probe with multi-band emission (pyrene, perylene, etc.) and introducing surfactant aggregates to tune their fluorescence emission. In our first try, we prepared a binary fluorescent ensemble based on a neutral bispyrene-based fluorophore and cationic surfactant dodecyltrimethylammonium bromide (DTAB) assemblies (Fan et al., 2015). The presence of DTAB assemblies in different aggregation states could efficiently adjust the emission of bispyrene probe from excimer-dominated emission to monomer-excimer co-emission until to monomer-dominated emission (Figure 13A). Protein sensing studies illustrated that the ensemble with monomer-excimer co-emission could exhibit ratiometric responses to non-metalloproteins and quenching responses to metalloproteins. The collection of fluorescence variations at monomer and excimer emission wavelengths could generate different recognition patterns to negatively-charged non-metalloproteins, positively-charged non-metalloproteins and metalloproteins, and then realize the differentiation among the three types of proteins (Figure 13B). The ratiometric responses to non-ometalloproteins were attributed to protein-induced further aggregation of the binary ensemble, while the turn-off responses to metalloproteins were ascribed to the energy/electron transfer from the ensemble to proteins. We also found the polarity of the spacer between the two pyrene units played important roles in the process of realizing cross-reactive sensing of proteins.

**Figure 14:**
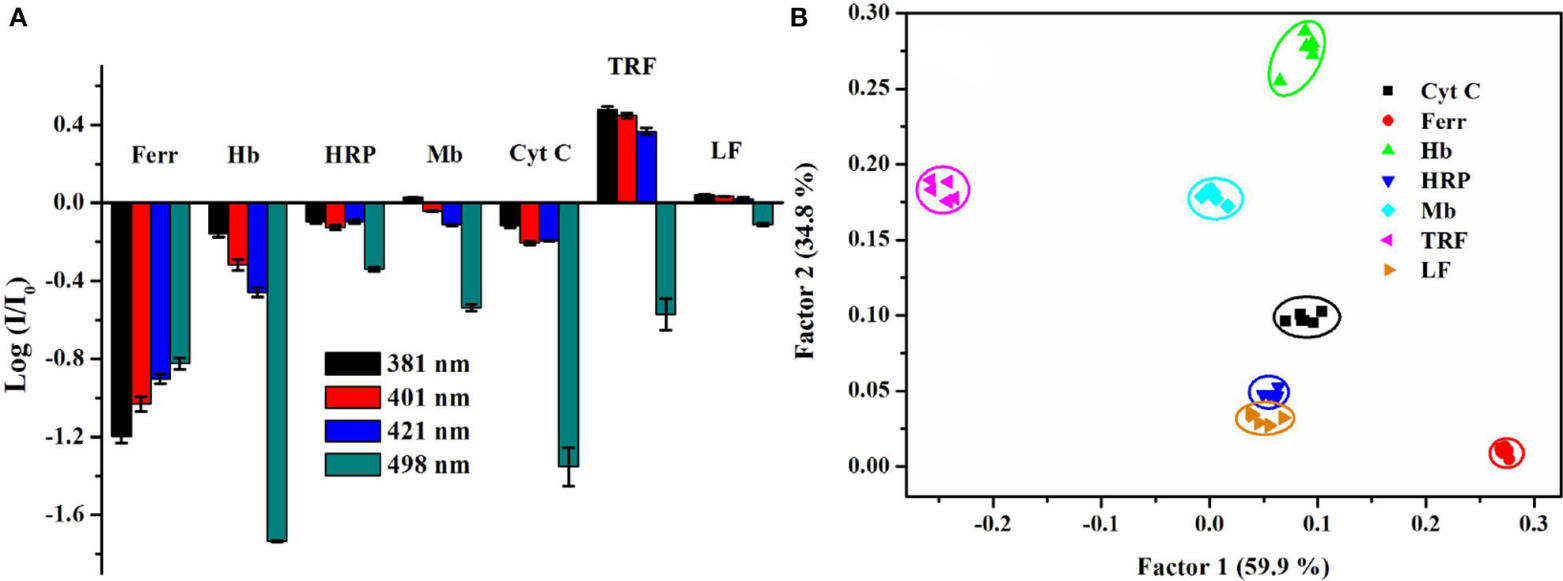
**(A)** Recognition patterns for different types of metalloproteins. **(B)** PCA score plot for identifying metalloproteins at 0.5 μM. Reprinted from Zheng et al. (2017) with permission.

In order to further identify the proteins belong to the same type, we developed a mini sensor array (Cao et al., 2016) and a single-sensor based discriminative system, which realized the identification of metalloproteins (Zheng et al., 2017). In the single-sensor system, our group have synthesized a mono-pyrene derivative containing a pyridine boronic acid unit to provide glycoprotein receptors and positive charges and a cholic acid group with self-assembly ability to increase the modulation effect by surfactant. The anionic surfactant, sodium dodecylbenzene sulfonate (SDBS), could well modulate the probe to emit multi-band emission. Besides, the selected binary ensemble provided unique recognition patterns for seven metalloproteins including glycoproteins by collecting four emission wavelengths (381, 401 nm for pyrene monomer emission, 421 nm for distorted excimer emission, and 498 nm for perfect excimer emission) (Figure 14A), and it showed good discrimination capability of proteins by PCA (Figure 14B). What's more, this ensemble sensor can also well distinguish proteins in serum and urine, suggesting there is great potential for practical applications.

To check the feasibility of the multi-wavelength cross-reactive strategy in identifying both non-metalloproteins and metalloproteins, we have designed a dual-fluorophore probe (bispyrene-modified perylene derivative) to provide more emission bands and signals. The cationic CTAB assemblies could effectively regulate the fluorescence emission of this dual-fluorophore probe from pyrene monomer emission to pyrene monomer-perylene co-emission (Bo et al., 2019). Just as we expected, the binary ensemble based on the dual-fluorophore probe and CTAB assemblies can provide fluorescence variation signals at six relative emission wavelengths (pyrene and perylene), which can generate a specific recognition pattern for a particular protein. Indeed, this single sensor can realize discrimination of four non-metalloproteins, four metalloproteins, unknown samples, and protein mixtures by LDA. In this study, the combination of a dual-fluorophore with multiple emission bands and the modulation effect of surfactant assemblies endows stronger discrimination ability for such binary ensemble sensors for proteins.

In addition, our group also synthesized an amphiphilic cholic acid-modified pyrene derivative, and found that it could form spherical aggregates and emit multiple fluorescence emission bands in aqueous solution (Fan et al., 2018). This fluorescent assembly showed ratiometric selective response to BSA or HSA among non-metalloproteins due to the disassembly of probe aggregates. But it showed turn-off response to four metalloproteins attributed to either electron or energy transfer from PyECA to bound metalloproteins. The different binding interactions of sensor platform and metalloproteins and the different contributions of two quenching mechanisms (electron or energy transfer) may be the reasons for the cross-reactive responses. This novel strategy is simple and can be extended to detect other analytes by using different supramolecular self-assemblies. Compared with multi-channel sensors, this type of sensors has outstanding advantages in data acquisition, because it only needs one scan of the emission spectrum to obtain the corresponding information. However, the biggest challenge of multi-band emission sensors is the high dependence on molecular design, which requires the introduction of multiple fluorophores or one fluorophore with multi-band emission.

## Conclusions and Outlook

In this review, we focused on different optical sensor arrays for protein discrimination and their applications in real samples. The three strategies for constructing such sensor arrays including multi-element-based sensor arrays, environment-sensitive sensor arrays and multi-wavelength-based single sensing systems for protein discrimination were described in detail. Besides, we tried to explain the connections and differences among different strategies.

Each type of array has its own advantages and disadvantages. For a multi-element-based sensor array, a series of sensor elements are usually required, and sometimes the number of sensor elements is even more than the number of analytes, making such arrays more complicated and time-consuming in the process of construction and data collection. But the recognition ability of this type of sensor array is easy to improve by increasing the sensor elements. The environment-sensitive sensor array is constructed by adjusting pH, polarity, viscosity, etc., and the multi-wavelength-based single system is based on an array of channels or emission bands, resulting in less dependent on design and synthesis of various probes (only one probe) compared with multi-element-based sensor arrays. Moreover, the third strategy using one single sensor system could significantly decrease sample consumption and simplify the data collection process. By comparison, the three types of sensor arrays have their own drawbacks. The construction and sensing process of the first type of array is the most time-consuming and costly, the second type of array is the most environment-sensitive and the least expandable, and the third type has highest requirements for the molecule design and instruments.

Although these three types of array-based sensors are different, they are closely related. For example, quantum dots (QDs) are involved in all three types but play different roles. In the first type, a variety of QDs are mainly used as sensor elements. But the second type involves only one type of QDs and the sensor elements are constructed by changing pH and ionic strengths. For the third type, the simplest one, the discrimination is achieved by collecting the signal changes of one type of QDs at different channels. It seems to be repetitive and unreasonable, but they actually serve their respective classifications very well.

Up to now, multi-element-based sensor arrays for protein identification have been boomingly developed, especially arrays constructed by nanomaterials as building blocks. The ability to realize strong discrimination ability of this type of sensor arrays based on as few as sensor elements is the big challenge and the future development trend. There have been some reports that realized using only two or three elements to achieve high-throughput detection of proteins. The other two types of optical biosensors have been relatively slowly developed. The challenge for the second type of environment-sensitive ones is the deep understanding of photophysical properties of a particular probe on the environments and design of new structural and effective probes. The development for the third type of multiple-wavelength cross-reactive single-system is highly dependent on the molecular design, which makes this type of sensor array grow slowly. But these two types also have broad application prospects because of easy preparation and simple data collection process. Particularly, the third type is more attractive due to the easy data collection and less consumption of samples. The aid of supramolecular assemblies on modulating photophysical properties of fluorescent probes makes this strategy more feasible. This type of optical sensor arrays will become a hot trend for developing discriminative sensors for proteins and other analytes.

## Author Contributions

JF and LQ designed and wrote the manuscript. HH and LD revised the manuscript. All authors listed have made a substantial, direct and intellectual contribution to the work, and approved it for publication.

## Conflict of Interest

The authors declare that the research was conducted in the absence of any commercial or financial relationships that could be construed as a potential conflict of interest.
